# Coronavirus Disease and New-Onset Atrial Fibrillation: Two Cases

**DOI:** 10.7759/cureus.8066

**Published:** 2020-05-12

**Authors:** Mohamed E Taha, Wail Alsafi, Moutaz Taha, Ammar Eljack, Hisham Ibrahim

**Affiliations:** 1 Internal Medicine, University of Iowa Hospitals and Clinics, Iowa City, USA; 2 Cardiovascular Medicine, Howard University, Washington, DC, USA; 3 Internal Medicine, Omdurman Islamic University, Khartoum, SDN; 4 Internal Medicine, University of Nevada Reno School of Medicine, Reno, USA; 5 Internal Medicine, Beaumont Health, Dearborn, USA; 6 Internal Medicine, University of Iowa Hospitals and Clinics, Iowa City, USA

**Keywords:** covid-19, atrial fibrillation, electrocardiogram, palpitations, severe acute respiratory syndrome coronavirus 2

## Abstract

Severe acute respiratory syndrome coronavirus 2 (SARS-CoV-2) infection has resulted in a considerable amount of morbidity and mortality worldwide since December 2019. Patients with coronavirus disease (COVID-19) most commonly present with respiratory manifestations, while cardiac manifestations were reported as a complication and seldom as a presenting feature. We report two cases of new-onset atrial fibrillation occurring in middle-aged men with no significant past medical history. The first patient presented with symptomatic atrial fibrillation; however, during his hospitalization course, he developed a fever, which led to the diagnosis of infection with SARS-CoV-2. The second patient presented from urgent care after being diagnosed with COVID-19 associated with newly diagnosed atrial fibrillation. Both patients were treated symptomatically for COVID-19 and discharged home after reverting to sinus rhythm. Physicians should be aware of the variable clinical presentations of COVID-19, especially in new or worsening cardiac illnesses, in order to practice the appropriate personal protection practices. More studies are needed to identify the viral mechanisms leading to the dysregulation of cardiac rhythm.

## Introduction

Coronavirus disease (COVID-19) is a current pandemic with high morbidity and mortality. The most common clinical features at presentation were viral prodromal and respiratory in nature, and cardiac manifestations are rarely reported as a presenting manifestation [1]. Cardiac manifestations typically develop as complications following the development of respiratory illness and include arrhythmias and acute myocardial injuries [2,3]. Early recognition of a COVID-19 infection regardless of the presenting clinical feature is imperative in preventing the transmission of the disease among the health care personnel and the community.

## Case presentation

Case 1

A 53-year-old white man with no significant past medical history presented to our facility with palpitations, dyspnea on exertion, and fatigue. He reported waking up at 2:00 am with sudden onset palpitation and shortness of breath upon minimal exertion, in addition to fatigue. There was no associated chest pain, cough, fevers, chills, sore throat, nausea, vomiting, or urinary symptoms on presentation, nor a history of previous similar symptoms. His symptoms persisted for six hours leading him to present to the emergency department (ED).

**Figure 1:**
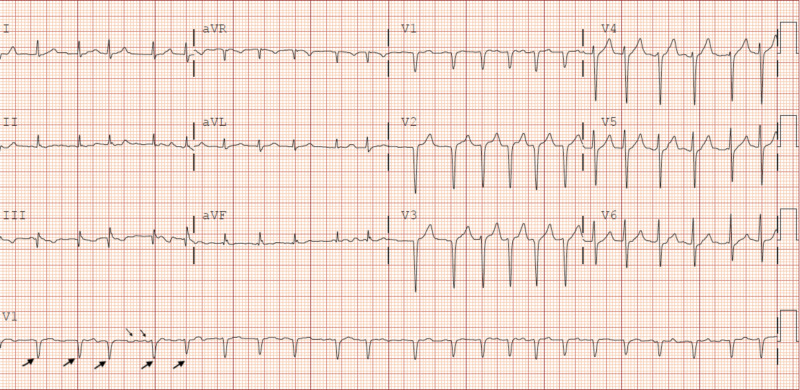
A 12-lead electrocardiogram showing atrial fibrillation with a pulse rate of 134 bpm with abnormal R wave progression in anterior leads.

Physical examination on presentation revealed irregular tachycardia with pulse rate (PR) 136 beats/minute (bpm), blood pressure (BP) 134/90 mmHg, respiratory rate (RR) 16 breaths/minute, oxygen saturation 96%, temperature 37.3°C, and body mass index (BMI) 30.7 kg/m^2^. The findings from the rest of his physical exam were unremarkable. An initial 12-lead electrocardiogram (ECG) revealed atrial fibrillation with PR 134 bpm and abnormal R wave progression (Figure 1).

**Figure 2:**
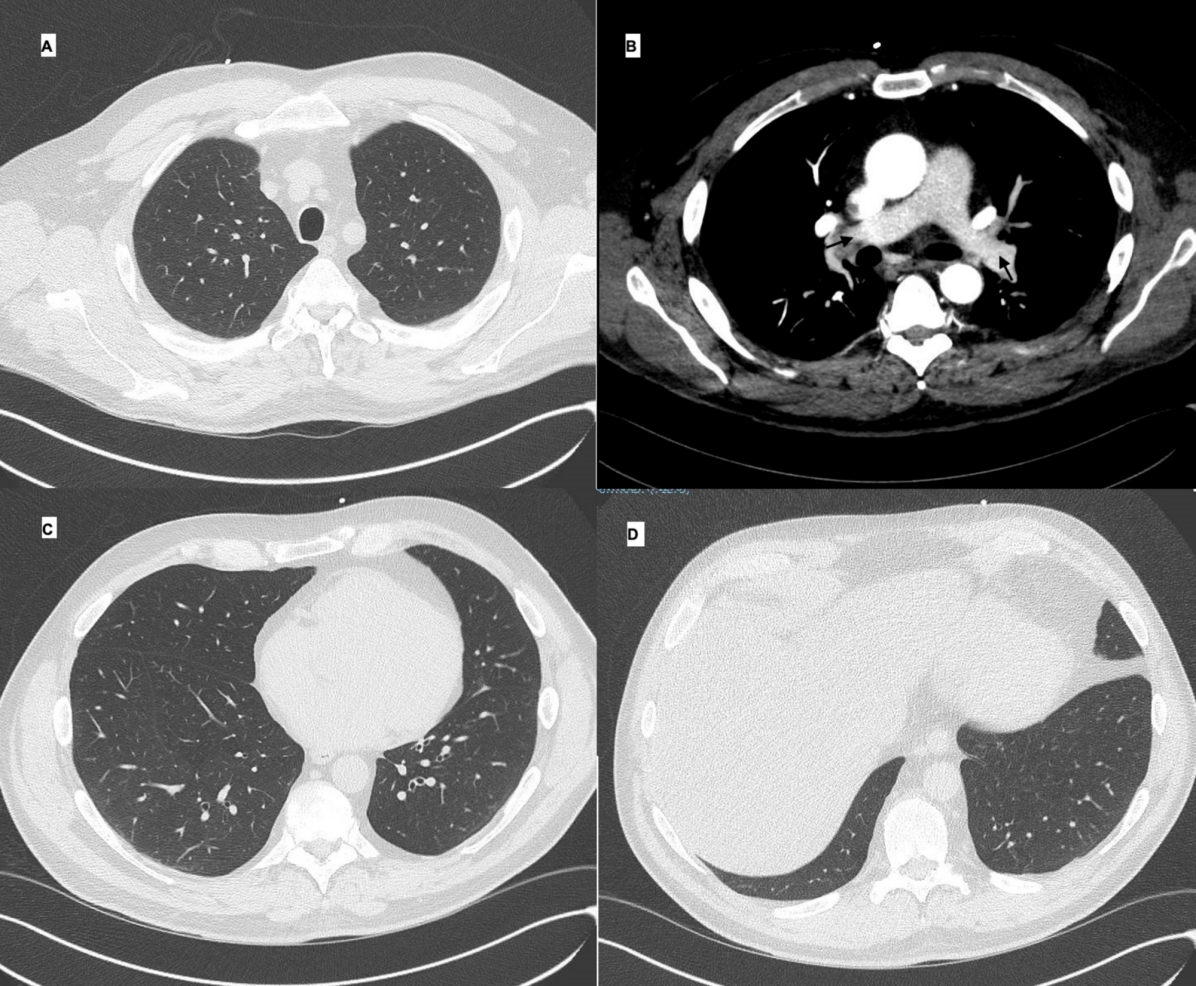
Computed tomography angiography of the chest showing no evidence of pulmonary embolism in major pulmonary arteries (B) and normal lung parenchyma (A, C, and D).

Laboratory tests revealed an elevated N-terminal (NT)-pro hormone brain natriuretic peptide (NT-proBNP) of 1.834 pg/dl (0-134 pg/dl), and a D-Dimer of 0.57 µg/ml (<0.50 µg/ml). Other laboratory results, including troponin T, thyroid-stimulating hormone value (TSH), complete blood count (CBC), comprehensive metabolic panel (CMP), and glycated hemoglobin (HbA1c), were unremarkable. A computed tomography angiogram of the chest was unremarkable (Figure 2).

He was treated with two doses of intravenous diltiazem (25 mg each) in the ED, which slowed his PR (to 90 from 99 bpm). He was also started on oral diltiazem (30 mg, every six hours) and admitted for observation. An echocardiogram performed the next morning revealed moderate left ventricular hypertrophy (LVH), left ventricular ejection fraction of 60%, healthy left atrial and ventricular size, and no other abnormalities.

Despite titrating up his diltiazem dose, his fatigue and dyspnea on exertion continued. His PR ranged from 90 to 110 bpm, increasing significantly with ambulation to over 130 bpm. A decision was made to pursue cardioversion after performing a transesophageal echocardiogram prior to discharge. However, that same evening he developed a fever (38.6°C) while his fatigue worsened. He was screened for sepsis and tested for severe acute respiratory syndrome-coronavirus 2 (SARS-CoV-2), and while he was not septic, he was positive for SARS-CoV-2 and diagnosed with coronavirus disease (COVID-19). Special isolation including droplet, contact, and eye protection in addition and symptomatic treatment with antipyretic Tylenol was initiated. The following morning, the patient spontaneously reverted to sinus rhythm, with dramatic improvement in his symptoms. He was discharged home and advised self-isolation with close outpatient follow-up monitoring.

Case 2

A 56-year-old Hispanic man with a past medical history of untreated essential hypertension presented to the ED with malaise and palpitations. He presented to urgent care the previous day with a one-week history of dry cough, low-grade fevers, chills, night sweats, and headaches, in addition to frequent palpitations. In urgent care, he tested positive for SARS-CoV-2. He was discharged home after prescribing symptomatic treatment and advised on home isolation.

**Figure 3:**
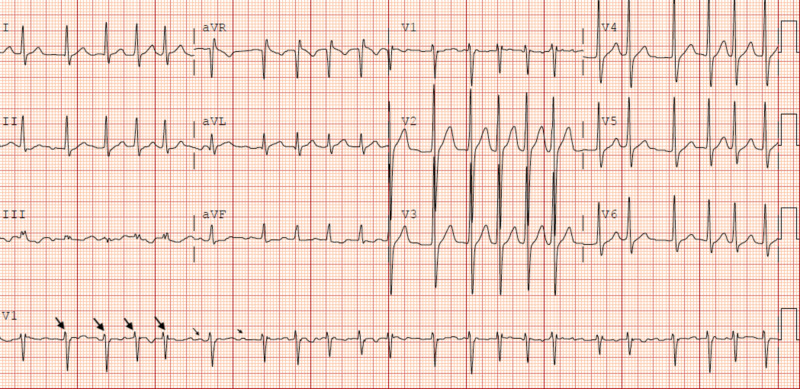
A 12-lead electrocardiogram showing atrial fibrillation with a pulse rate of 131 bpm and a borderline prolonged QT interval.

Upon presentation to the ED, we noted he was tachycardic with PR 130-140 bpm and febrile (38.8°C). His BP was 124/100 mmHg and RR 16 breaths/minute, with normal oxygen saturation on room air. His cardiopulmonary examination revealed irregular tachycardia with PR 144 bpm and left lower lobe crackles. No other systemic examination findings were remarkable. A 12-lead ECG revealed atrial fibrillation with PR 131 bpm along with features of LVH (Figure 3). 

**Figure 4:**
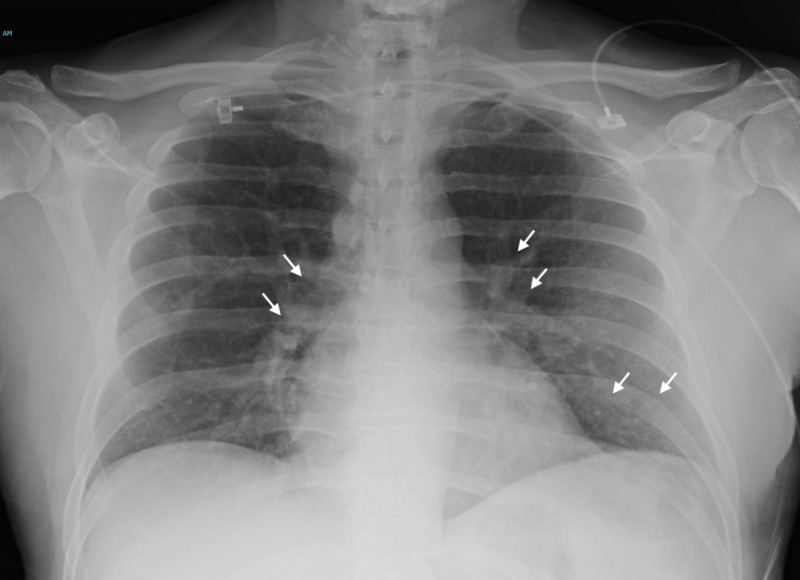
Chest x-ray showing left lower lobe infiltrates in addition to patchy left perihilar ground-glass airspace disease, and right middle lobe ground-glass airspace disease.

His chest x-ray revealed left lower lobe infiltrates in addition to patchy left perihilar and right middle lobe ground-glass airspace disease (Figure 4). 

Laboratory values, including CBC, CMP, TSH, troponin T, and HbA1c were only positive for mild lymphopenia; the other results were unremarkable. He was treated intravenous diltiazem (10 mg bolus) and a 500 ml normal saline intravenous fluid bolus, which led to an improvement in his PR to approximately 100 bpm. Thereafter, he was started on intravenous diltiazem drip at 5 mg/hour and admitted for monitoring and further workup. Additionally, he received symptomatic treatment with antipyretics given the fever. He was maintained in the appropriate droplet, and contact isolation with eye protection measures during his hospitalization course. 

## Discussion

COVID-19, a disease caused by SARS-CoV-2, was declared a pandemic by the World Health Organization in February 2020. SARS-CoV-2 is a beta coronavirus in the same subgenus as the SARS virus SARS-CoV as well as Middle East respiratory syndrome coronavirus (MERS-CoV). According to one recent study, the most common clinical features at presentation in COVID-19 patients were viral prodromal and respiratory, with fever as the most common (99% of cases), followed by fatigue (70%), dry cough (59%), anorexia (40%), myalgias (35%), dyspnea (31%), and sputum production (27%) [1]. Cardiac manifestations are rarely reported as a presenting manifestation; instead, cardiac manifestations typically develop as complications following the development of respiratory illness and include arrhythmias, acute myocardial injuries, myocarditis, late myocardial dysfunction, venous thromboembolism, and cardiopulmonary arrest with pulseless electrical activity or ventricular fibrillation during the recovery phase of pulmonary illness [2,3]. These manifestations, although rare, are considered a marker of a poor outcome as these patients tend to have more severe illness and a higher mortality rate [4]. Our first patient presented with new-onset atrial fibrillation (NOAF) with neither preceding viral prodrome nor associated respiratory concerns, while the other patient presented with NOAF on the background of initial mild viral prodrome and upper respiratory symptoms.

The pathophysiology of COVID-19 related cardiac manifestations including arrhythmias is not well understood, and proposed theories include direct viral myocardial damage, hypoxia, hypotension, enhanced inflammatory status, angiotensin-converting enzyme 2 receptor downregulation, electrolytes abnormalities in the acute phase of severe illness, drug toxicity, and endogenous catecholamine adrenergic status [5]. It was unclear why our two patients developed NOAF. However, an increased serum NT-proBNP levels and fevers could point to direct viral damage to the conductive system or cytokine-mediated injury as a result of an enhanced inflammatory state. Moreover, the presence of undiagnosed and untreated hypertension seemed to be a common risk factor in both cases.

The management of COVID-19 is mainly supportive and directed towards treating symptoms and complications, and no specific vaccine, antiviral drugs, or other agents are currently approved for COVID-19 [6]. There are currently no clear guidelines on the management of atrial fibrillation in COVID-19 patients; however, following the standard guidelines in treating atrial fibrillation with rapid ventricular response was sufficient in controlling the arrhythmias in our patients.

## Conclusions

NOAF could be a presenting feature or can occur early in the course of COVID-19, and usually responds to the standard treatment for atrial fibrillation. High levels of clinical suspicion are needed to recognize COVID-19 early, regardless of the presenting clinical feature, to prevent the transmission of the disease among the health care personnel and the community. Furthermore, additional studies are needed to identify the exact viral mechanisms leading to dysregulation of the cardiac rhythm and long-term monitoring to determine outcomes in these patients.
